# Esophageal bronchogenic cyst treated with submucosal tunneling endoscopic resection: two case reports

**DOI:** 10.1186/s13256-024-04453-y

**Published:** 2024-04-02

**Authors:** Hui Sha, Zong-Dan Jiang

**Affiliations:** 1 Department of Gastroenterology, The People’s Hospital of Yining, Xinjiang, China; 2 Department of Gastroenterology, Nanjing First Hospital, Nanjing Medical University, 68 Changle Road, Nanjing, 210006 Jiangsu China

**Keywords:** Esophageal bronchogenic cyst, Submucosal tunneling endoscopic resection, Endoscopic argon plasmacoagulation, Case report

## Abstract

**Introduction:**

Although esophageal bronchogenic cysts are benign diseases, they may be accompanied by serious complications and have the possibility of recurrence. Therefore, once confirmed, it is necessary to treat the esophagobronchial cyst when the contraindication is excluded. Endoscopic treatment is usually used for lesions with small diameter and shallow origin, and has the advantages of small surgical trauma and risk, which can reduce the psychological burden of patients to a certain extent, help them to recover quickly, and lower hospital costs.

**Case presentation:**

Case 1 is a 54-year-old Han Chinese man admitted to our hospital who complained of difficulty swallowing in the past 6 months. Case 2 is a 41-year-old Han Chinese man who was hospitalized in the past 3 months due to chest discomfort. Endoscopic ultrasound revealed a hypoechoic cystic lesion arising from the muscularis propria. Submucosal tunneling endoscopic resection was performed using a dual knife, and a cystic mass was observed between the mucosa and the muscular layers of the esophagus. On locating the cyst, an incision was made on the oral side of the lesion for evacuation. The cyst wall was excised using endoscopic argon plasma coagulation. We successfully removed the esophageal bronchogenic cyst lesion in the intrinsic muscle layer using submucosal tunneling endoscopic resection.

**Conclusion:**

Esophageal bronchogenic cysts are rare in clinical practice and lack specificity in clinical manifestations. Multiple methods can be used to determine the location and nature of the lesion and ultimately determine the treatment plan. Surgical resection and endoscopic treatment are two different treatment methods, and appropriate treatment plans need to be selected on the basis of the origin layer, size, and relationship with the esophagus of the lesion to reduce complications and improve prognosis.

## Introduction

Bronchogenic cyst is a rare benign congenital malformation that results from an abnormal budding of the primitive foregut. It has been reported to occur in the mediastinum, lung parenchyma, pericardium, thymus, and so on [1], but the esophageal type is rarely seen [2]. Bronchogenic cysts are usually asymptomatic. Symptoms may develop at any age due to the mass effect and the compression of mediastinal airways and the esophagus. Symptoms often include coughing, dyspnea, respiratory distress, and dysphagia. Although it is a benign lesion, it has the potential for malignant transformation, and the growth of the cyst may cause bleeding, infection, and even rupture [3, 4]. Normally, surgical resection is recommended for this disease. Thoracotomy and thoracoscopy are commonly used surgical methods [5]. Endoscopic treatment of esophageal duplication cysts has increased in recent years because of its minimal invasiveness [6, 7].

## Case presentations

### Patient 1

**Fig. 1:**
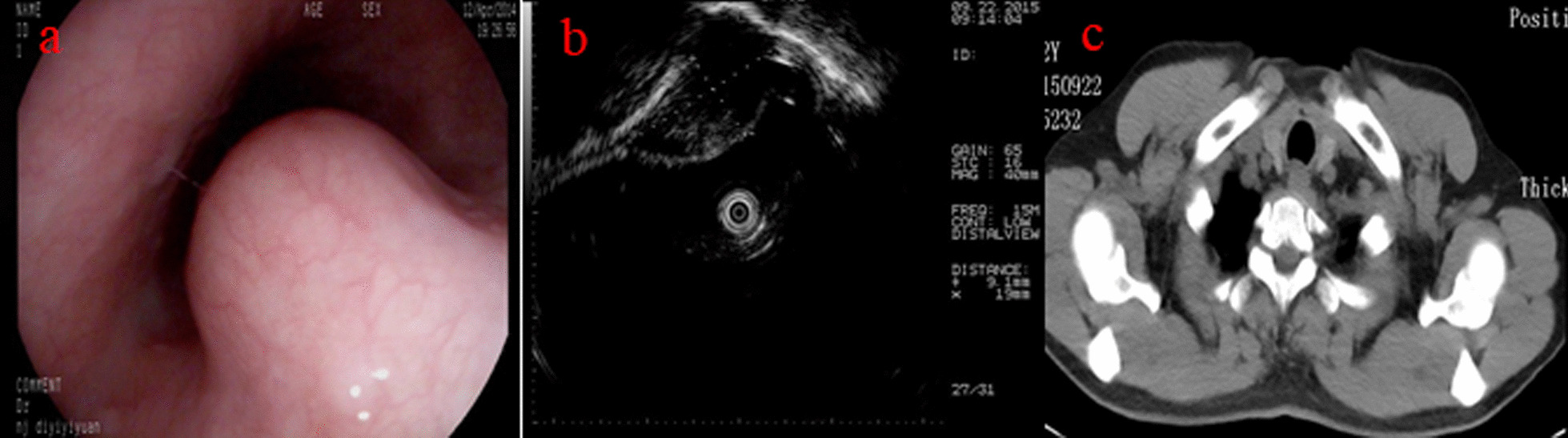
**a** Endoscopic image showing a 20 × 20 mm bulging lesion in the proximal esophagus. **b** Endoscopic ultrasound revealed a hypoechoic cystic lesion arising from the muscularis propria. **c** Computed tomography revealed an obvious mass located in the proximal esophagus

A 54-year-old Han Chinese man was admitted to our hospital with the complaint of difficulty swallowing for the previous 6 months in September 2015. He had not received any examination or treatment in the past 6 months. His past medical history was unremarkable. Physical examination did not reveal any obvious abnormal signs. Upper endoscopy showed a 20 × 20 mm bulging lesion in the proximal esophagus (Fig. 1a). Endoscopic ultrasound showed a 9.1 × 19 mm hypoechoic cystic lesion arising from the muscularis propria (Fig. 1b). A well-defined cystic mass was observed on computed tomography (CT) (Fig. 1c).

**Fig. 2:**
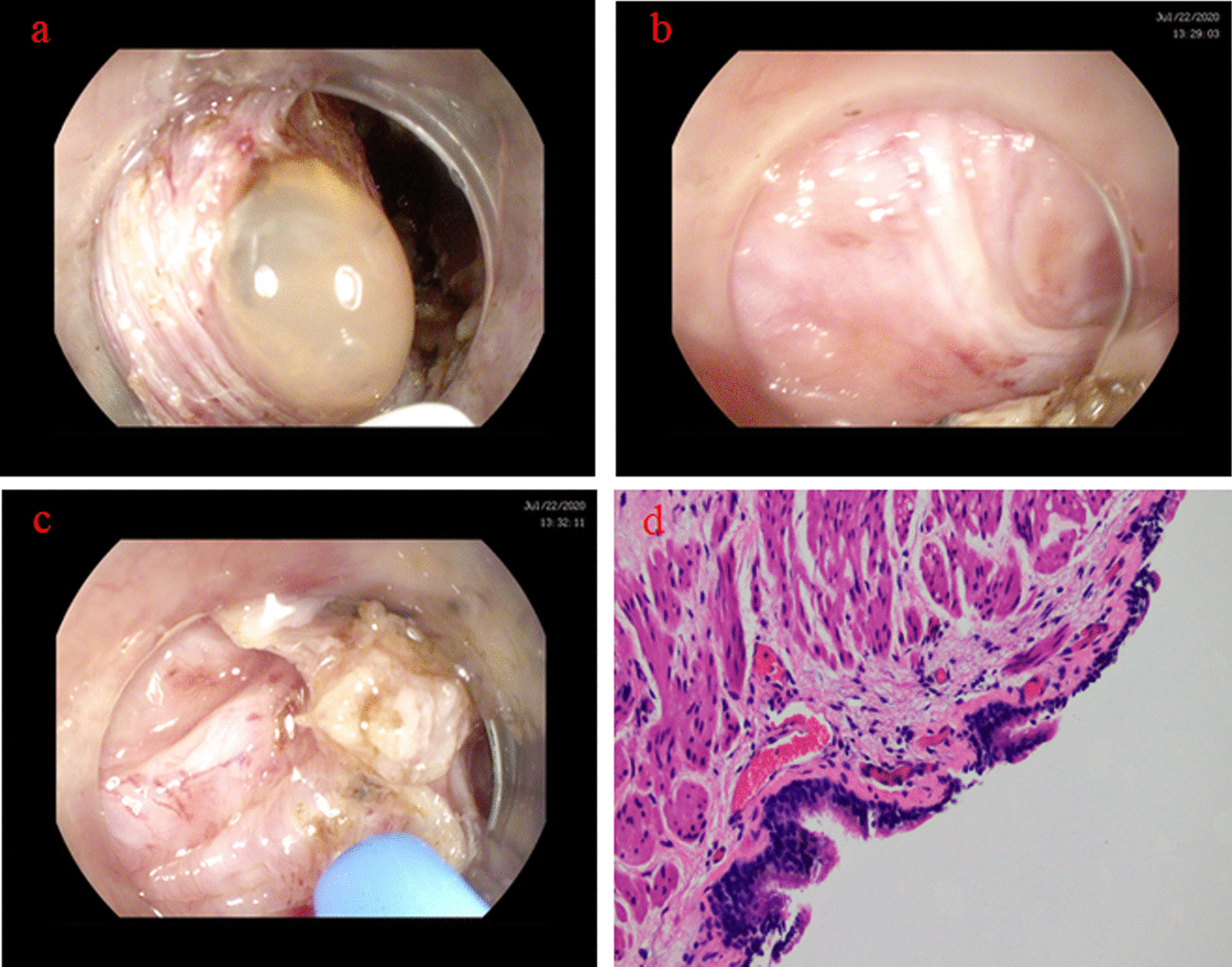
**a** Cyst fluid spraying out after incision. **b** Internal view of the cyst wall was demonstrated. **c** Cyst wall was excised using endoscopic argon plasma coagulation. **d** Microscopic findings showed the cyst wall tissue lined by ciliated columnar epithelium

On the third day of admission, submucosal tunneling endoscopic resection (STER) was performed using a dual knife (KD-650L, Olympus, Tokyo, Japan), and a cystic mass was observed between the mucosa and the muscular layers of the esophagus. On locating the cyst, an incision was made on the oral side of the lesion for evacuation. A small amount of yellowish fluid sprayed out and an intact cyst was shown under endoscopy (Fig. 2a, b). The cyst wall was excised using endoscopic argon plasma coagulation (Fig. 2c). Histopathological examination showed a cuboidal epithelium lined cyst wall, which contained cartilage and a few bronchial glands, consistent with a bronchogenic cyst (Fig. 2d).

### Patient 2

**Fig. 3:**
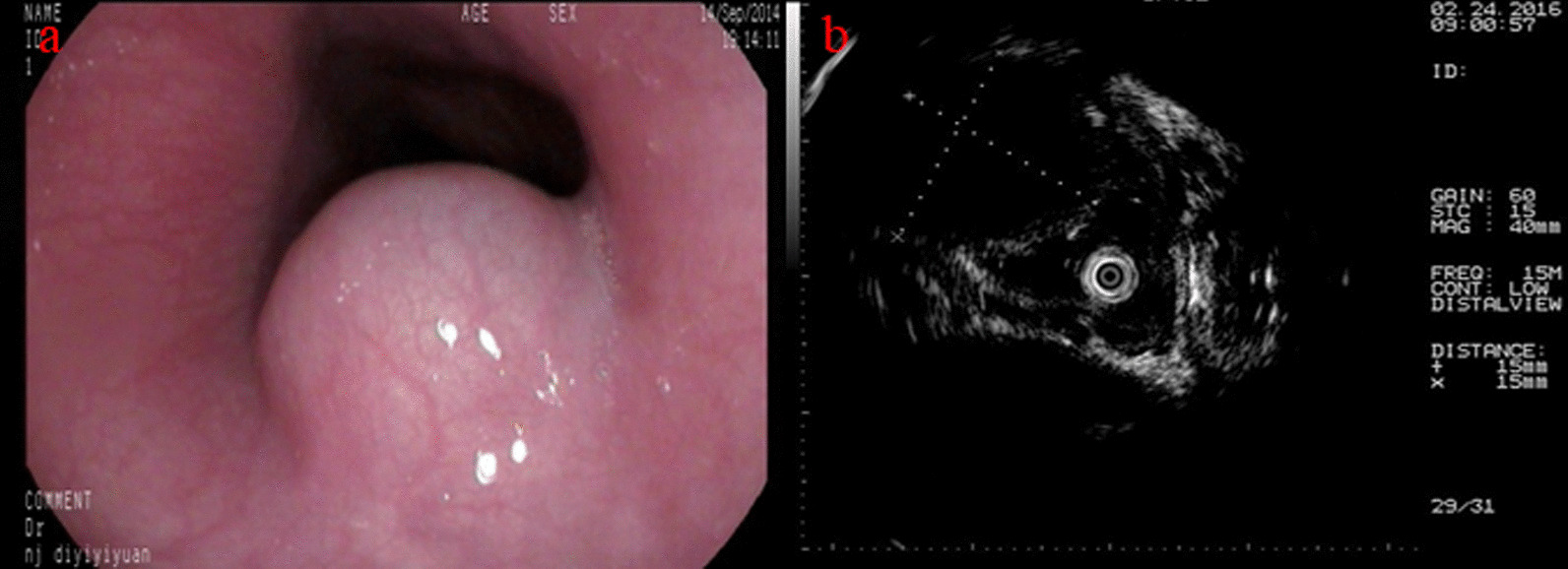
**a** Endoscopic image showing a 15 × 15 mm bulging lesion in the esophagus 30 cm from the incisors. **b** Endoscopic ultrasound revealed a hypoechoic cystic lesion arising from the muscularis propria

A 41-year-old Han Chinese man was admitted to our hospital with the complaint of chest discomfort for the previous 3 months in February 2016. He had not received any examination or treatment in the past 3 months. The patient’s family history and past medical history were both unremarkable. Physical examination also did not reveal any obvious abnormal signs. Upper endoscopy showed a 15 × 15 mm bulging lesion in the esophagus 30 cm from the incisors (Fig. 3a). Endoscopic ultrasound showed a 15 × 15 mm hypoechoic cystic lesion arising from the muscularis propria (Fig. 3b).

**Fig. 4:**
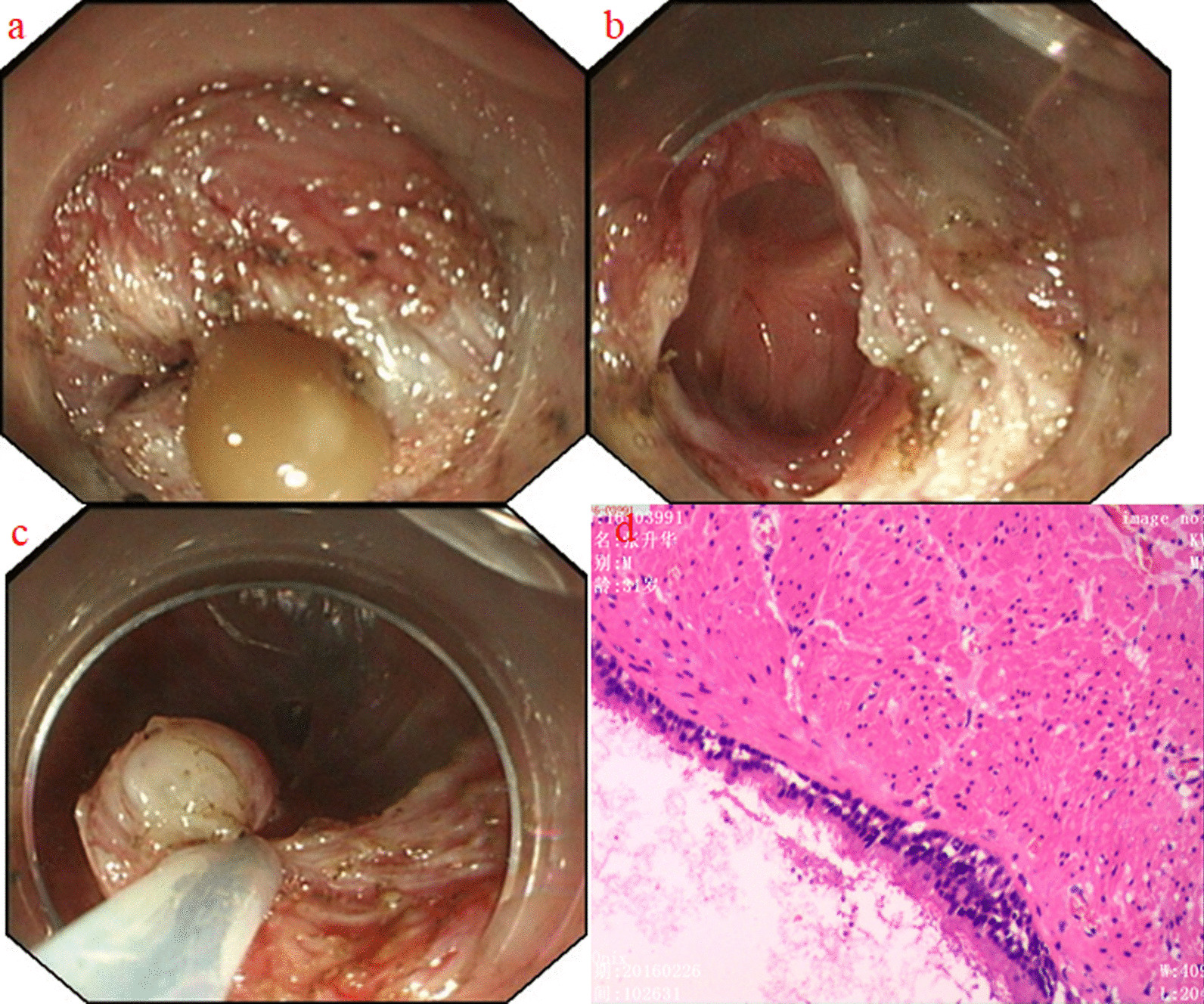
**a** Cyst fluid spraying out after incision. **b** Internal view of the cyst wall was demonstrated. **c** The snare removed part of the cyst wall for pathological examination. **d** Microscopic findings showed the cyst wall tissue lined by ciliated columnar epithelium

On the third day of admission, we performed STER using a dual knife (KD-650L, Olympus, Tokyo, Japan), and a cystic mass was observed between the mucosa and the muscular layers of the esophagus. On locating the cyst, an incision was made on the oral side of the lesion for evacuation. An amount of yellowish fluid sprayed out and an intact cyst was shown under endoscopy (Fig. 4a, b). The snare removed part of the cyst wall for pathological examination (Fig. 4c). Histopathological examination showed that there was a cystic cavity in the smooth muscle of esophagus, which was lined with a single layer of ciliated columnar epithelium, and there was red stained liquid in the cystic cavity. The morphology was consistent with the esophageal submucosal bronchogenic cyst (Fig. 4d).

## Discussion

Esophageal bronchogenic cyst is a rare disease first reported by Maier in 1948 [8]. It is usually located in the right posterior lower mediastinum and is a congenital mass caused by abnormal sprouting of the trachea and bronchi [9]. The cyst wall is composed of bronchial components, namely bronchial cartilage, ciliated columnar epithelium, and smooth muscles. Due to its secretory function and inability to excrete, the endothelium of bronchogenic cysts can become increasingly enlarged. Although the disease often starts from a young age, some patients have no obvious symptoms in the early stages and gradually develop symptoms as they age, resulting in patients being diagnosed at an older age.

Esophageal bronchogenic cyst is often diagnosed as esophageal benign tumor such as leiomyoma before operation. In the past, X-ray and CT scans were the two most valuable diagnostic methods [10], but these tests did not differentiate between benign and malignant tumors, and further examination is therefore needed. Akutsu *et al*. [11] proposed that fluorodeoxyglucose-positron emission tomography (FDG-PET) is an effective diagnostic tool for distinguishing bronchial cysts from malignant tumors. With the development of endoscopic technology, endoscopic ultrasound (EUS) has also become an important differential diagnostic tool. It is sensitive enough as to distinguish between cystic and solid masses, and can clearly describe the size, origin layer, and relationship between the cyst and the esophagus, which helps to diagnose esophageal lesions [12]. In addition, EUS fine needle aspiration (EUS-FNA) is also an important method for diagnosing cystic lesions [13]. The differential diagnosis between esophageal and bronchial cysts and repetitive cysts is more challenging, and its diagnosis requires pathological reports. EUS-FNA can obtain pathological specimens.

Resection in adults is usually performed by surgical or thoracotomy. Complications such as acute inflammation, ulcerations, infections, and fistulization affect its application. In our two cases, we used STER for treatment of the esophageal bronchogenic cyst. The submucosal tunneling technique was originally developed as a submucosal endoscopy with a mucosal flap safety valve [14]. It was found to be feasible for natural orifice transluminal endoscopic surgery (NOTES), and subsequently developed for per oral endoscopic myotomy (POEM) in the treatment of esophageal achalasia [15–17]. In POEM, an esophageal mucosal incision is made to create a submucosal tunnel that crosses the gastroesophageal (GE) junction, thus providing a submucosal space to operate in. This ultimately led to the development of STER [18]. STER is a method for treating submucosal tumors arising from the muscularis propria. Studies have shown that STER is effective and safe in removing submucosal tumors of the esophagus and cardia. Compared with surgery, STER may be a less complex and risky option, which provides a minimally invasive approach, especially for patients with higher surgical risks [19]. After performing STER, we cut the wall of the cyst, and then destroy the epithelial cell layer of the cyst wall by using endoscopic argon plasmacoagulation. Theoretically, the recurrence rate of cysts may be lower, but further studies on the long-term efficacy of this treatment are required.

## Conclusion

For esophageal cystic lesions, preoperative EUS can better evaluate the origin of the lesion, differentiate whether it is cystic or solid, and measure the size of the cyst. If the cyst is located within the wall, endoscopic resection can be considered. Lesions located in the intrinsic muscle layer can be treated using the STER method. We successfully removed the esophageal bronchogenic cyst lesion in the intrinsic muscle layer using STER. The surgery was minimally invasive and safe, with fast recovery and follow-up.

## Data Availability

The datasets used and/or analyzed during the current study are available from the corresponding author on reasonable request.
